# Microbial functional genes elucidate environmental drivers of biofilm metabolism in glacier-fed streams

**DOI:** 10.1038/s41598-017-13086-9

**Published:** 2017-10-04

**Authors:** Ze Ren, Hongkai Gao, James J. Elser, Qiudong Zhao

**Affiliations:** 1 Flathead Lake Biological Station, University of Montana, Polson, MT 59860 USA; 2 State Key Laboratory of Cryosheric Sciences, Northwest Institute of Eco-Environment and Resources, Chinese Academy of Sciences, Lanzhou, 730000 China; 3 School of Life Sciences, Arizona State University, Tempe, AZ 85281 USA; 4 School of Geography and Planing, Sun Yat-Sen University, Guangzhou, 510275 China

## Abstract

Benthic biofilms in glacier-fed streams harbor diverse microorganisms driving biogeochemical cycles and, consequently, influencing ecosystem-level processes. Benthic biofilms are vulnerable to glacial retreat induced by climate change. To investigate microbial functions of benthic biofilms in glacier-fed streams, we predicted metagenomes from 16s rRNA gene sequence data using PICRUSt and identified functional genes associated with nitrogen and sulfur metabolisms based on KEGG database and explored the relationships between metabolic pathways and abiotic factors in glacier-fed streams in the Tianshan Mountains in Central Asia. Results showed that the distribution of functional genes was mainly associated with glacier area proportion, glacier source proportion, total nitrogen, dissolved organic carbon, and pH. For nitrogen metabolism, the relative abundance of functional genes associated with dissimilatory pathways was higher than those for assimilatory pathways. The relative abundance of functional genes associated with assimilatory sulfate reduction was higher than those involved with the sulfur oxidation system and dissimilatory sulfate reduction. Hydrological factors had more significant correlations with nitrogen metabolism than physicochemical factors and anammox was the most sensitive nitrogen cycling pathway responding to variation of the abiotic environment in these glacial-fed streams. In contrast, sulfur metabolism pathways were not sensitive to variations of abiotic factors in these systems.

## Introduction

Glaciers provide vital water resources^1–3^ and harbor complex microbial communities^4–6^, particularly in arid regions in Central Asia^7–10^. However, as a dynamic interface between the cryosphere, hydrosphere, atmosphere, pedosphere, and biosphere, glacial ecosystems are extremely susceptible to ongoing climate change^11–14^. Glacier-fed streams integrate upstream catchment processes and constitute a prominent geomorphological and ecological component of the alpine landscape^14,15^. In glacier-fed streams, the proportional contribution of ice-melt and snowmelt to overall stream flow diminishes with increasing distance from the glaciers^11,16,17^. This shifting relationship dramatically influences the stream habitat template, including water temperature, channel stability, conductivity, and solute chemistry^16,18–20^. Accordingly, hydrological alteration associated with glacial retreat has the potential to re-structure the habitat template of glacier-fed streams with consequences for the associated biota, including benthic microbial communities and their ecosystem functions.

The highly variable environments of glacier-fed streams impose strong controls on benthic biota^16,21^. Spatial and temporal variation in glacier runoff affect aquatic community structure and biodiversity^11,22^, while nutrient availability influences the growth of microorganisms and alters their community structure^23,24^. More specifically, the microbial diversity of benthic biofilms increases with distance from the source glacier, and declines with glacier area proportion, glacier source proportion, and elevation^14,24^. Importantly, this diversity decreases with glacial retreat, illustrating the sensitivity of benthic microbial communities to the effects of climate change^14^.

Microorganisms drive the bulk of biogeochemical cycles on the planet and thus influence ecosystem-level functioning^25^. Functional traits are valuable ecological markers to understand microbial community assembly^26^ and help elucidate how natural communities and their functions respond to environmental changes^27,28^. In streams, for example, biofilms are hot spots of microbial activity^29^, contributing substantially to metabolism and biogeochemical cycles through nutrient uptake, transfer of nutrients to higher trophic levels, and remineralization^30–33^. Unraveling the functional potential of microbial assemblages is critical for gaining insights into their roles in ecosystem processes and their response to environment change^34,35^. For example, microbial metabolism involves a large set of functional genes and biochemical pathways that power biogeochemical cycling in ecosystems. Microbial nitrogen metabolic processes such as nitrification, denitrification, and nitrogen uptake drive nitrogen cycling in streams, controlling nitrogen exports to downstream^36–38^. In anaerobic environments, sulfur compounds are major electron carriers for anaerobic microbial metabolism and sulfate reduction is an important process in organic matter degradation^39–41^. However, our understanding of microbial functions of benthic biofilm in glacier-fed streams remains extremely limited.

In Central Asia, glacial ecosystems are experiencing unprecedented global warming, hydrological variation, and glacier retreat rapidly^42–45^. Glacier-fed stream ecosystems in this semi-arid and cold region are particularly vulnerable. Understanding ecosystem functions and predicting the impacts of climate change on glacier-fed stream ecosystems calls for much better knowledge about microbial processes. High throughput molecular technologies and advanced bioinformatics tools make it possible to predict the functional capacity of microbial communities. Following up on our previous study of microbial communities in glacier-fed streams^24^, this research explores the predicted functions of microbial assemblages in benthic biofilms and their relationships to hydrological and physicochemical factors in glacier-fed streams. We predicted metagenomes from 16s rRNA gene sequence data using PICRUSt (Phylogenetic Investigation of Communities by Reconstruction of Unobserved States)^46^, identified functional genes associated with nitrogen and sulfur metabolisms based on KEGG (Kyoto Encyclopedia of Genes and Genomes) database^47^, and explored the relationships between metabolic pathways and abiotic factors. We hypothesized that the functional genes of biofilm community in glacier-fed streams are closely associated with community taxonomic compositions and abiotic environments, especially hydrological factors. Although metagenomics reflects potential rather than realized functional capacity, our data offer a window into the poorly known metabolism of benthic biofilms in glacier-fed streams, providing insights into the potential impacts of glacial melting on the ecosystem function.

## Methods

### Study Area

The Tianshan Mountains are located in Central Asia. Based on the dataset from the Second Glacier Inventory of China^48^, there are 7934 glaciers distributed in the China’s Tianshan Mountain with a total area of 7179 km^2^ and an ice volume of 707 km^3^, which account for 16.3%, 13.9%, and 15.7% of the total number, total area, and total volume of glaciers in China, respectively^49^. With global warming, these glaciers have been shrinking rapidly^42–45^. For example, the Urumqi Glacier No. 1 (GN1, 43°06′N, 86°49′E) in eastern Tianshan Mountain had an area of 1.95 km^2^ and a terminus altitude of 3730 m in 1962, but by 2010 had shrunk to 1.65 km^2^ and the terminus retreated to the altitude of 3743 m^50^.

### Sampling

**Figure 1:**
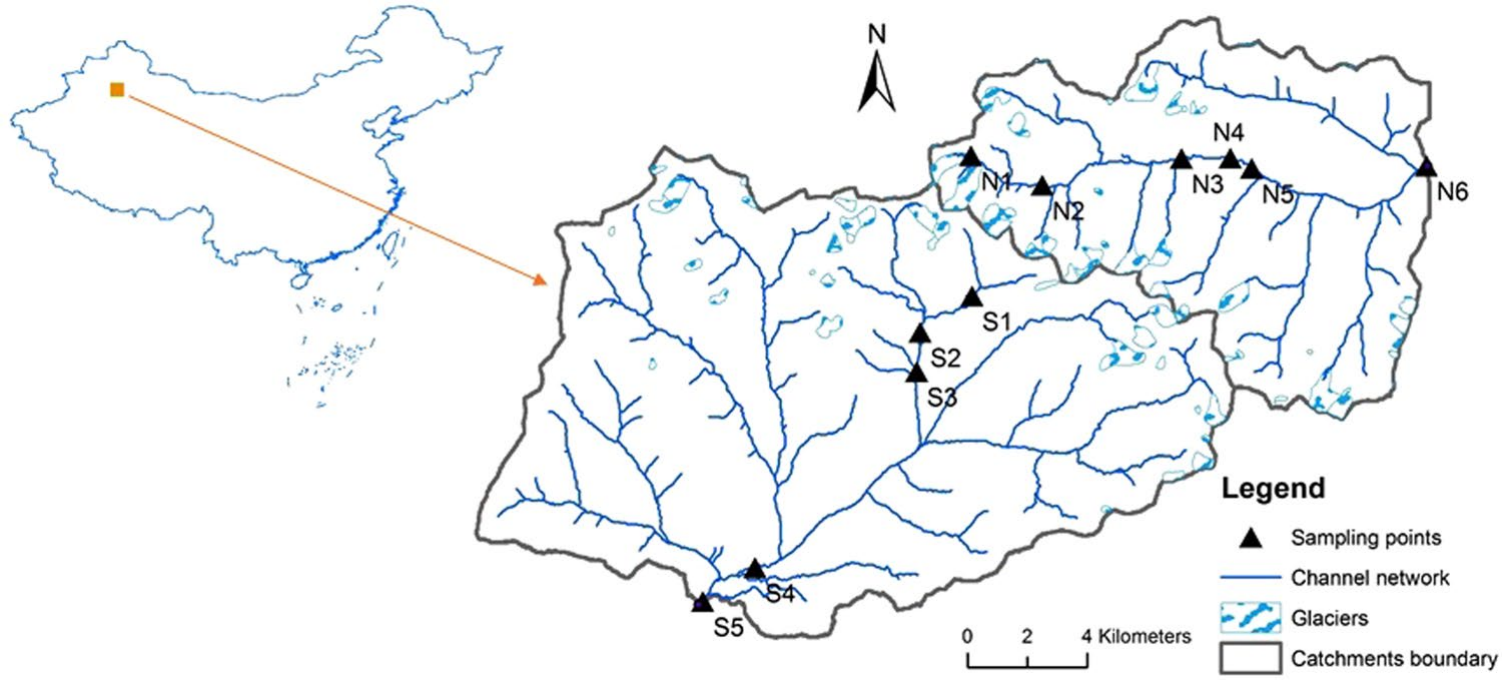
Study area and sample sites. 11 samples were collected from two glacier-fed streams in June 2016 in the Tianshan Mountains, Central Asian. The map is from Ren *et al*. (2017)^24^ and was created in ArcGIS 14.0 (http://desktop.arcgis.com/en/arcmap/) using DEM image download from USGS (https://earthexplorer.usgs.gov/).

In June 2016, we collected water and benthic biofilm samples from 11 sample sites distributed along two glacier-fed streams in the Tianshan Mountains (Fig. 1). The samples from the sites S1-S5 (elevations ranged from 3548 to 2840 m) were collected on June 19, 2016. The samples from the sites N1-N6 (elevations ranged from 3828 to 2646 m) were collected on June 20, 2016. At each sample site, we randomly sampled 6–9 submerged rocks from the river cross section at depths of 10 to 30 cm. The benthic biofilm was removed by rigorously brushing an area of 4.5 cm diameter from the upper surface of each stone using a sterilized nylon brush (changed between samples) and rinsing the slurry with sterile water. From the mixed slurry, approximately 10 ml was filtered through a 0.2-μm membrane filter that was immediately frozen in liquid nitrogen in the field and transported frozen to the lab after all the sampling work. In the lab, benthic biofilm samples were stored at −80 °C until DNA extraction.

### DNA Extraction, PCR, and Sequencing

Bacterial 16S rRNA genes were analyzed to determine the predicted functional genes and metabolic pathways of the microbial community in biofilms. DNA extraction, PCR, and sequencing were described in our previous study^24^.

### Physicochemical factors

At each sample site, water temperature (Temp), dissolved oxygen (DO), pH, and conductivity (Cond) were measured *in situ* using a YSI handheld meter (Model 80, Yellow Springs Instruments, Yellow Springs, Ohio). Elevation was measured using a GPS unit (Triton 500, Magellan, Santa Clara, California). Water samples were collected for nutrients and dissolved organic carbon (DOC) analysis. Total nitrogen (TN) was analyzed by ion chromatography after persulfate oxidation (EPA 300.0)^51^. Nitrate (NO_3_^−^) was determined by ion chromatography (EPA 300.0)^51^. Ammonium (NH_4_^−^) was analyzed using the indophenol colorimetric method (EPA 350.1)^52^. Total phosphorus (TP) was quantified using the ammonium molybdate method after oxidation (EPA 365.3)^53^. Soluble reactive phosphorus (SRP) was analyzed using the ammonium molybdate method (EPA 365.3)^53^. DOC was analyzed using a Shimadzu TOC Analyzer (TOC-VCPH, Shimadzu Scientific Instruments, Columbia, Maryland). The results of these basic physicochemical factors are shown in our previous study^24^.

### Hydrological Factors

The glacier distribution data were obtained from the Second Glacier Inventory of China^48^. The river channel network and the catchment boundaries were derived from topographic data, the Digital Elevation Model (DEM) at 90 m resolution (https://earthexplorer.usgs.gov/). Hydrological factors were calculated using the landscape-based hydrological model of Gao *et al*. (2016, 2017)^10,17^, including the glaciated area proportion (G_A_) for each sample site, runoff proportion generated from glacier source (G_S_), and distance to glaciers (G_D_). Methods for calculating these basic hydrological factors are shown in our previous study^24^.

### Analysis

Bacterial 16s rRNA sequence data (available at National Center for Biotechnology Information, SRP115356) were cleaned using the software package QIIME^54^ and then clustered to operational taxonomic units (OTUs) with a complete linkage algorithm on a 97% sequence identity level. The metagenomes were predicted from 16s data using Phylogenetic Investigation of Communities by Reconstruction of Unobserved States (PICRUSt)^46^. The functional genes associated with nitrogen metabolism and sulfur metabolism were identified based on Kyoto Encyclopedia of Genes and Genomes (KEGG) database^47^. Redundancy analysis (RDA)^55^ was conducted to reveal the association of microbial communities in relation to abiotic factors based on functional gene abundances using vegan package 2.4^56^ in R 3.3.2^57^. A Monte Carlo test was used to assess the significance of abiotic factors in RDA^55^. Mantel tests were run to assess how functional dissimilarity (Bray-Curtis distance calculated from functional gene abundances) correlated with taxonomic dissimilarity (Bray-Curtis distance calculated from taxon abundances), environmental distance (Euclidean distance), and geographic distance. Correlation analysis was conducted to assess the relationships between metabolic pathways and abiotic factors with the P-value adjusted by Bonferroni correction using psych package 1.7.5^58^ in R 3.3.2^57^.

## Results and Discussion

### Functional Variation

**Figure 2:**
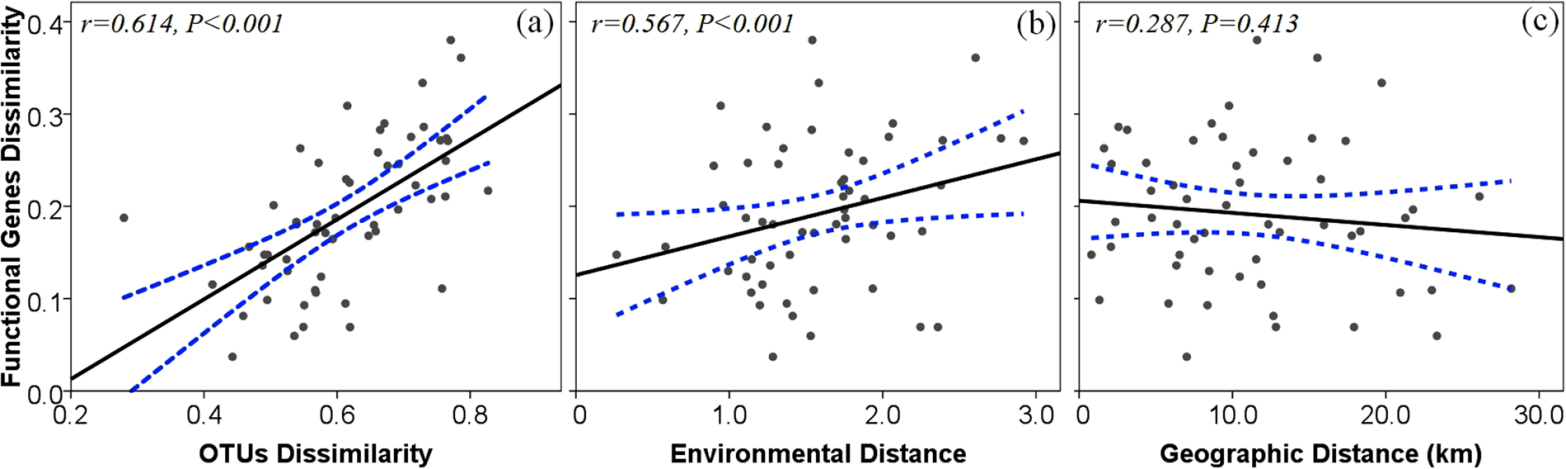
Plots of Mantel tests showing the relationships between (**a**) functional genes dissimilarity and OTUs dissimilarity, (**b**) functional genes dissimilarity and environmental distance, and (**c**) functional genes dissimilarity and geographic distance. One point represents one sample pair. Blue dotted lines denote the 95% confidence interval.

**Figure 3:**
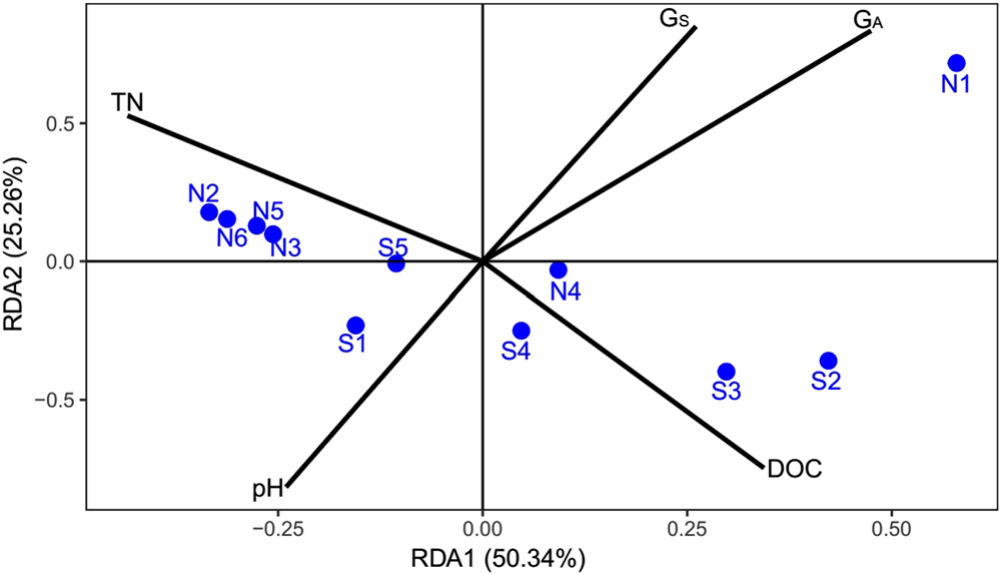
RDA plot revealing the association of microbial communities (based on functional gene abundances) in relation to abiotic factors. The first and second axes accounted for 75.6% of the variance (RDA 1: 50.34%; RDA 2: 25.26%). Only the abiotic factors that were significantly correlated with the community (forward selection with Monte Carlo test, P < 0.05) are shown as the black solid lines. Blue dots represent the sampling sites and the abbreviations are the names of the sampling sites.

The 16S rRNA data set consisted of 127,146 sequences clustered in 7545 OTUs and 5771 functional genes. The Bray-Curtis distance calculated from functional gene abundances was significantly correlated with operational taxonomic units (OTUs) dissimilarity (Bray-Curtis distance calculated from taxon abundances, Mantel r = 0.614, P < 0.001) and environmental distance (Euclidean distance, Mantel r = 0.567, P < 0.001), but not with geographic distance (Mantel r = 0.287, P = 0.413, Fig. 2), suggesting that the overall changes in functional genes composition were closely associated with community taxonomic composition and abiotic conditions. Redundancy analysis (RDA) was applied to depict the spatial distribution of microbial communities in relation to effects of physicochemical and hydrological factors in terms of the functional gene composition (Fig. 3). The first two axes accounted for 75.6% of the variance (RDA 1: 50.34%; RDA 2: 25.26%). A Monte Carlo test showed that glacier area proportion (G_A_), glacier source proportion (G_S_), total nitrogen (TN), dissolved organic carbon (DOC), and pH were the abiotic factors most closely associated (p < 0.05) with the distribution of microbial communities (Fig. 3). Canonical correspondence analysis based on taxon abundances showed similar results^24^.

Different biomes clearly harbor distinct microbial assemblages^27,59,60^. It has been demonstrated that metagenomic functional composition is strongly correlated with taxonomic composition in spite of functional redundancy at the genomic level^61,62^. Moreover, recent work also suggests that the metabolic functional potential of microbial communities in ocean and soil is closely related with environmental conditions^63^. Our study also indicates that the functional metabolic potential of glacier-fed streams is strongly influenced by environmental conditions and taxonomic structure.

### Potential Nitrogen Metabolism

Nitrogen has various chemical species that are cycled by a suite of coupled biogeochemical processes^64^ that are catalyzed by various microbe-derived enzymes^65,66^. To deepen our understanding of glacier-fed stream ecosystems, it is crucial to know which N cycling pathways are operating and how they respond to the surrounding abiotic environment. In this study component, we identified and compared the potential nitrogen cycle pathways that might occur in the glacier-fed streams based on the functional genes encoding the enzymes required for specific nitrogen cycling pathways.

**Figure 4:**
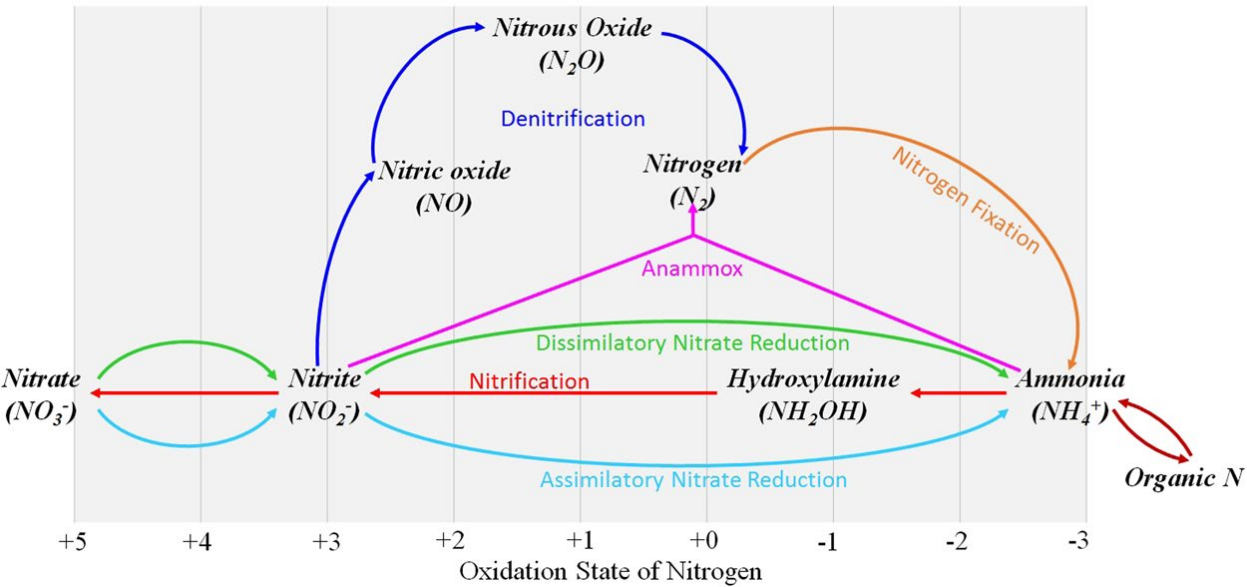
A simplified schematic of the nitrogen cycle constructed with the use of the KEGG nitrogen metabolism pathway. The N cycle consists of interconnected biogeochemical pathways (dissimilatory nitrate reduction, assimilatory nitrate reduction, denitrification, nitrogen fixation, nitrification, and anammox). There are a variety of genes encoding enzymes that catalyze the important transformation reactions of various nitrogen forms ranging from the oxidation states +5 in nitrate to −3 in ammonium. The percentage of associated gene reads were predicted using PICRUSt based on KEGG database.

**Figure 5:**
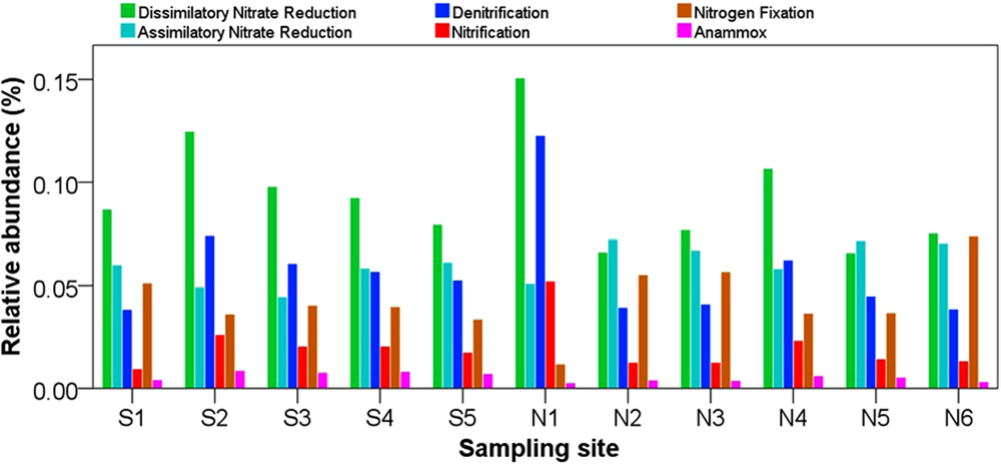
Relative abundance of major categories of functional genes encoding the enzymes that catalyze nitrogen cycling pathways (dissimilatory nitrate reduction, assimilatory nitrate reduction, denitrification, nitrification, nitrogen fixation, and anammox) based on the KEGG database.

Of the 5,571 detected genes, 48 functional genes were associated with nitrogen metabolism (Figure S1) and the relative abundance of these functional genes ranged from 0.79% to 0.89% of the whole gene reads. Figure S1 summarizes the relative abundance of the genes encoding enzymes that catalyze nitrogen metabolism. The N cycle involves four reduction pathways (assimilatory nitrate reduction, dissimilatory nitrate reduction to ammonia, denitrification, and nitrogen fixation) and two oxidation pathways (nitrification and anammox) (Fig. 4). The relative abundance of genes associated with assimilatory nitrate reduction and dissimilatory nitrate reduction ranged from 0.044% to 0.072% and from 0.066% to 0.151%, respectively (Fig. 5). The assimilatory pathways require energy and start with the reduction of NO_3_^−^ to NO_2_^−^ and then to NH_4_^+^, which is highly bioavailable and can be readily used by cells for synthesis of amino acids and nucleotides^67,68^. Dissimilatory nitrate reduction to ammonia uses N-compounds to provide energy (ATP) and is another catabolic pathway that can retain the nitrogen in the system in a bioavailable form (NH_4_^+^) for further biological processes^69,70^. Generally, assimilatory pathways were much more prevalent than dissimilatory pathways^71^. In our studied glacier-fed streams, however, genes associated with dissimilatory pathways were much more abundant that those involved in assimilatory pathways, suggesting that the microbial assemblages in glacier-fed streams use inorganic nitrogen more as an energy source more as an N source for biosynthesis. Nitrogen fixation is an energetically demanding process and only a few taxonomic groups genera can carry out this process^72,73^. As a competing process to nitrate reduction pathways, denitrification is the main biological process for removal of N from freshwater systems^70,74^. The relative abundance of genes associated with denitrification and nitrogen fixation ranged from 0.038% to 0.123% and from 0.012% to 0.074%, respectively (Fig. 5). Nitrification is an essential process in nitrogen cycle performed by nitrifiers converting ammonium to nitrate. In our study, functional genes encoding enzymes that catalyze nitrification had a relative abundance ranges from 0.01% to 0.05% (Fig. 5). Anammox plays a significant role in the nitrogen cycle, converting nitrite and ammonia to dinitrogen^75^, and is another microbial process that releases fixed nitrogen from the environment as dinitrogen^76–79^. In our study, functional genes encoding enzymes that catalyze anammox had only a very small relative abundance (Fig. 5).

In ecosystems, nitrogen metabolic pathways are particularly susceptible to environmental redox fluctuations because of the large difference between the oxidation state of nitrate (+5) in nitrate and that of ammonia (−3)^80,81^. Moreover, some other environmental factors also strongly impact nitrogen cycling. For example, microorganisms can be stimulated by high concentration of NO_3_^−^ to increase populations containing the functional genes encoding the enzymes required for nitrate reduction. Low N:P ratios and low NH_4_^+^ can stimulate N_2_ fixing bacteria^82–84^. It has also been demonstrated that anammox is influenced by physicochemical properties, such as nitrate concentration, C/N ratio, ammonia concentration, and pH^85^. Nitrification can be affected by temperature, salinity, light, organic matter concentration, substrate concentrations, pH, and oxygen concentration^86^. In our study, we explored the relationships between nitrogen cycle pathways and abiotic environment including physicochemical and hydrological factors (Table S1). The results showed that anammox was negatively correlated with G_A_ and G_S_. Denitrification, dissimilatory nitrate reduction, and nitrification were negatively correlated with G_D_. In addition, anammox was positively correlated with pH, temperature, and DOC but negatively correlated with TN and NO_3_. Assimilatory nitrogen reduction was positively correlated with TN and NO_3_. Nitrification was negatively correlated with pH. Nitrogen fixation was positively correlated with NH_4_. These results suggest that hydrological factors had more significant influences on nitrogen metabolism than physicochemical factors. Among the nitrogen cycling pathways, anammox was the most sensitive to variation of abiotic environment in the glacial-fed streams we studied (Table S1). Since global warming leads to significant changes of abiotic environment, especially in the hydrology in glacier-fed streams in Central Asia, these results may allow us to predict changes in nitrogen metabolism under climate change.

### Potential Sulfur Metabolism

**Figure 6:**
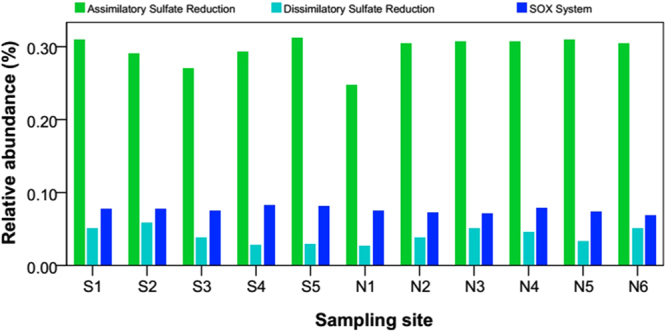
Relative abundance of major categories of functional genes encoding the enzymes that catalyze sulfur metabolism, including assimilatory sulfate reduction, dissimilatory sulfate reduction, and the SOX system.

Overall, there were 43 functional genes associated with sulfur metabolism in our data set (Figure S2). The relative abundance of these functional genes ranged from 0.848% to 1.009% of the total gene reads. Figure S2 summaries the relative abundance of the genes encoding sulfur metabolism based on the KEGG database. Assimilatory sulfate reduction occurs in many anoxygenic phototrophic bacteria and leads to the biosynthesis of sulfur-containing compounds^87,88^. Dissimilatory sulfate reduction is a pathway in which sulfate or sulfur serves as the terminal electron acceptor of the respiratory chain, producing inorganic sulfide. In our study, the relative abundance of genes associated with assimilatory sulfate reduction ranged from 0.247% to 0.313%, followed by genes involved in the sulfur oxidation (SOX) system and in dissimilatory sulfate reduction (Fig. 6), suggesting that microbial assemblages in glacier-fed streams regard inorganic sulfur as an S source in biosynthesis more than an electron acceptor. The SOX system, a sulfur oxidation pathway, is a multienzyme pathway found in both photosynthetic and lithoautotrophic sulfur-oxidizing bacteria^39,89,90^. Sulfur oxidation is a critical step in the biogeochemical sulfur cycle^91,92^. Our detection of genes encoding the enzymes catalyzing sulfur oxidation suggests that microbial activity is generating acidity that may also affect mineral formation and stability^88^. Previous studies have suggested that the availability of electron donors^93^, light^94^, and aerobic conditions^95^ are important environmental factors affecting sulfur metabolism. In our study, only assimilatory sulfate reduction was positively correlated with G_D_ (Table S1), suggesting that sulfur metabolism pathways were not sensitive to variations of hydrological and physicochemical factors in glacier-fed streams.

## Conclusions

In glacier-fed streams, microbial assemblages in benthic biofilms change significantly with variation in the abiotic environment and thus appear vulnerable to glacier retreat caused by climate change. As most glaciers on Earth are receding rapidly, the habitat template (especially hydrological factors) of glacier-fed streams will be increasingly disrupted. Community taxonomic composition and the abiotic environment regulate microbe-mediated functions. In particularly, hydrological factors have significant impacts on the distribution of functional genes and nitrogen cycling pathways. Thus, large changes in biogeochemical processes and associated ecosystem functions likely will occur as glacier-fed ecosystems are transformed due to loss of their association with the cryosphere.

## Electronic supplementary material

Supplementary Information

## References

[1] Barnett TP, Adam JC, Lettenmaier DP (2005). Potential impacts of a warming climate on water availability in snow-dominated regions. Nature..

[2] Gardner AS (2013). A reconciled estimate of glacier contributions to sea level rise: 2003 to 2009. Science..

[3] Zemp M (2015). Historically unprecedented global glacier decline in the early 21st century. J. Glaciol..

[4] Schuette UME (2010). Bacterial diversity in a glacier foreland of the high Arctic. Mol. Ecol..

[5] Liu Q, Zhou Y, Xin Y (2015). High diversity and distinctive community structure of bacteria on glaciers in China revealed by 454 pyrosequencing. Syst. Appl. Microbiol..

[6] Wilhelm L (2014). Rare but active taxa contribute to community dynamics of benthic biofilms in glacier-fed streams. Environ. Microbiol..

[7] Yao T, Pu J, Lu A, Wang Y, Yu W (2007). Recent glacial retreat and its impact on hydrological processes on the tibetan plateau, China, and sorrounding regions. Arct. Antarct. Alp. Res..

[8] Sorg A, Bolch T, Stoffel M, Solomina O, Beniston M (2012). Climate change impacts on glaciers and runoff in Tien Shan (Central Asia). Nat. Clim. Change..

[9] Duethmann D, Menz C, Jiang T, Vorogushyn S (2016). Projections for headwater catchments of the Tarim River reveal glacier retreat and decreasing surface water availability but uncertainties are large. Environ. Res. Lett..

[10] Gao H, Ding Y, Zhao Q, Hrachowitz M, Savenije HHG (2017). The importance of aspect for modelling the hydrological response in a glacier catchment in Central Asia. Hydrol. Process..

[11] Brown LE, Hannah DM, Milner AM (2007). Vulnerability of alpine stream biodiversity to shrinking glaciers and snowpacks. Global Change Biol..

[12] Finn DS, Raesaenen K, Robinson CT (2010). Physical and biological changes to a lengthening stream gradient following a decade of rapid glacial recession. Global Change Biol..

[13] Jacobsen D, Milner AM, Brown LE, Dangles O (2012). Biodiversity under threat in glacier-fed river systems. Nat. Clim. Change..

[14] Wilhelm L, Singer GA, Fasching C, Battin TJ, Besemer K (2013). Microbial biodiversity in glacier-fed streams. Isme J..

[15] Robinson CT, Tonolla D, Imhof B, Vukelic R, Uehlinger U (2016). Flow intermittency, physico-chemistry and function of headwater streams in an Alpine glacial catchment. Aquat. Sci..

[16] Milner AM, Brown LE, Hannah DM (2009). Hydroecological response of river systems to shrinking glaciers. Hydrol. Process..

[17] Gao, H., Han, T., Liu, Y. & Zhao, Q. Use of auxiliary data of topography, snow and ice to improve model performance in a glacier-dominated catchment in Central Asia. *Hydrology Research*. h2016242 (2016).

[18] Hannah DM (2007). Integrating climate-hydrology-ecology for alpine river systems. Aquat. Conserv..

[19] Kuhn J (2011). Spatial variability in macroinvertebrate assemblages along and among neighbouring equatorial glacier-fed streams. Freshwater Biol..

[20] Milner AM, Brittain JE, Castella E, Petts GE (2001). Trends of macroinvertebrate community structure in glacier-fed rivers in relation to environmental conditions: a synthesis. Freshwater Biol..

[21] Robinson CT, Thompson C, Freestone M (2014). Ecosystem development of streams lengthened by rapid glacial recession. Fund. Appl. Limnol..

[22] Cauvy-Fraunie S, Espinosa R, Andino P, Jacobsen D (2015). and Dangles, O. Invertebrate Metacommunity Structure and Dynamics in an Andean Glacial Stream Network Facing Climate Change. PLoS One..

[23] Kohler TJ, Van Horn DJ, Darling JP, Takacs-Vesbach CD, Mcknight DM (2016). Nutrient treatments alter microbial mat colonization in two glacial meltwater streams from the McMurdo Dry Valleys, Antarctica. FEMS Microbiol. Ecol..

[24] Ren Z, Gao HK, Elser JJ (2017). Longitudinal variation of microbial communities in benthic biofilms and association with hydrological and physicochemical conditions in glacier-fed streams. Freshwater Sciences..

[25] Kuang J, Huang L, He Z, Chen L, Hua Z (2016). Predicting taxonomic and functional structure of microbial communities in acid mine drainage. Isme J..

[26] Barberan A, Fernandez-Guerra A, Bohannan BJM, Casamayor EO (2012). Exploration of community traits as ecological markers in microbial metagenomes. Mol. Ecol..

[27] Fierer N (2012). Cross-biome metagenomic analyses of soil microbial communities and their functional attributes. P. Natl. Acad. Sci. Usa..

[28] Green JL, Bohannan BJM, Whitaker RJ (2008). Microbial biogeography: From taxonomy to traits. Science..

[29] Geesey GG, Mutch R, Costerton JT, Green RB (1978). Sessile bacteria: an important component of the microbial population in small mountain streams. Limnol. Oceanogr..

[30] Schiller DV, Marti E, Riera JL, Sabater F (2007). Effects of nutrients and light on periphyton biomass and nitrogen uptake in Mediterranean streams with contrasting land uses. Freshwater Biol..

[31] Battin TJ, Kaplan LA, Newbold JD, Hansen C (2003). Contributions of microbial biofilms to ecosystem processes in stream mesocosms. Nature..

[32] Buchkowski RW, Schmitz OJ, Bradford MA (2015). Microbial stoichiometry overrides biomass as a regulator of soil carbon and nitrogen cycling. Ecology..

[33] Van Horn DJ, Sinsabaugh RL, Takacs-Vesbach CD, Mitchell KR, Dahm CN (2011). Response of heterotrophic stream biofilm communities to a gradient of resources. Aquat. Microb. Ecol..

[34] Freedman ZB, Zak DR (2015). Atmospheric N deposition alters connectance, but not functional potential among saprotrophic bacterial communities. Mol. Ecol..

[35] Wang, K. *et al*. Regional variations in the diversity and predicted metabolic potential of benthic prokaryotes in coastal northern Zhejiang, East China Sea. *Sci. Rep.-UK*. **6** (2016). 10.1038/srep38709 PMC5137025 27917954

[36] Peterson BJ (2001). Control of nitrogen export from watersheds by headwater streams. Science..

[37] Zeglin, L. H. Stream microbial diversity responds to environmental changes review and synthesis of existing research. *Front. Microbiol*. **6**, 10–3389 (2015). 10.3389/fmicb.2015.00454 PMC4435045 26042102

[38] Mulholland PJ (2008). Stream denitrification across biomes and its response to anthropogenic nitrate loading. Nature..

[39] Koizumi Y, Kojima H, Fukui M (2005). Potential sulfur metabolisms and associated bacteria within anoxic surface sediment from saline meromictic Lake Kaiike (Japan). FEMS Microbiol. Ecol..

[40] Overmann J, Beatty JT, Krouse HR, Hall KJ (1996). The sulfur cycle in the chemocline of a meromictic Salt Lake. Limnol. Oceanogr..

[41] Jorgensen BB (1982). Mineralization of organic matter in the sea bed - the role of sulphate reduction. Nature..

[42] Liu Q, Liu S (2016). Response of glacier mass balance to climate change in the Tianshan Mountains during the second half of the twentieth century. Clim. Dynam..

[43] Wang P (2016). Comparison of changes in glacier area and thickness on the northern and southern slopes of Mt. Bogda, eastern Tianshan Mountains. J. Appl. Geophys..

[44] He Y (2015). Glacier variation in response to climate change in Chinese Tianshan Mountains from 1989 to 2012. J. Mt. Sci.-Engl..

[45] Aizen VB, Aizen EM, Kuzmichonok VA (2007). Glaciers and hydrological changes in the Tien Shan: simulation and prediction. Environ. Res. Lett..

[46] Langille MGI (2013). Predictive functional profiling of microbial communities using 16S rRNA marker gene sequences. Nat. Biotechnol..

[47] Kanehisa M, Goto S (2000). KEGG: kyoto encyclopedia of genes and genomes. Nucleic Acids Res..

[48] Guo, W. Q. *et al*. The Second Glacier Inventory Dataset of China (Version 1.0). Cold and Arid Regions Science Data Center at Lanzhou, (2014).

[49] Liu SY (2015). The contemporary glaciers in China based on the Second Chinese Glacier Inventory. Acta Geographica Sinica..

[50] Zhang G, Li Z, Wang W, Wang W (2014). Rapid decrease of observed mass balance in the Urumqi Glacier No. 1, Tianshan Mountains, central Asia. Quatern. Int..

[51] EPA 300.0, Determination of Inorganic Anions by Ion Chromatography. Environmental Monitoring Systems Laboratory, U.S. Environmental Protection Agency, Cincinnati, Ohio (1993).

[52] EPA 350.1, Determination of Ammonia Nitrogen by Semi-Automated Colorimetry. Environmental Monitoring Systems Laboratory, U.S. Environmental Protection Agency, Cincinnati, Ohio (1993).

[53] EPA 365.3, Phosphorous, All Forms (Colorimetric, Ascorbic Acid, Two Reagent). Environmental Monitoring Systems Laboratory, U.S. Environmental Protection Agency, Cincinnati, Ohio (1978).

[54] Caporaso JG (2010). QIIME allows analysis of high-throughput community sequencing data. Nat. Methods..

[55] Borcard, D., Gillet, F. and Legendre, P. Numerical Ecology with R. Springer New York. (2011).

[56] Oksanen, J. *et al*. vegan: Community Ecology Package. R package version 2.4-3. https://CRAN.R-project.org/package=vegan. (2017).

[57] R Core Team. R: A Language and Environment for Statistical Computing, R Foundation for Statistical Computing, Vienna, Austria. https://www.R-project.org. (2017).

[58] Revelle, W. psych: Procedures for Personality and Psychological Research, Northwestern University, Evanston, Illinois, USA, https://CRAN.R-project.org/package=psych. (2017).

[59] Hugerth LW (2015). Metagenome-assembled genomes uncover a global brackish microbiome. Genome Biol..

[60] Louca S, Parfrey LW, Doebeli M (2016). Decoupling function and taxonomy in the global ocean microbiome. Science..

[61] Gilbert, J. A. *et al*. The Taxonomic and Functional Diversity of Microbes at a Temperate Coastal Site: A ‘Multi-Omic’ Study of Seasonal and Diel Temporal Variation. *PLoS One*. **5**, (2010). 10.1371/journal.pone.0015545 PMC2993967 21124740

[62] Bryant JA, Stewart FJ, Eppley JM, Delong EF (2012). Microbial community phylogenetic and trait diversity declines with depth in a marine oxygen minimum zone. Ecology..

[63] Louca S (2016). *High tax*onomic variability despite stable functional structure across microbial communities. Nature Ecology & Evolution..

[64] Ollivier J (2011). Nitrogen turnover in soil and global change. FEMS Microbiol. Ecol..

[65] Falkowski PG, Fenchel T, Delong EF (2008). The microbial engines that drive Earth’s biogeochemical cycles. Science..

[66] Gruber N, Galloway JN (2008). An Earth-system perspective of the global nitrogen cycle. Nature..

[67] Richardson DJ, Watmough NJ (1999). Inorganic nitrogen metabolism in bacteria. Curr. Opin. Chem. Biol..

[68] Sparacino-Watkins C, Stolz JF, Basu P (2014). Nitrate and periplasmic nitrate reductases. Chem. Soc. Rev..

[69] Zumft WG (1997). Cell biology and molecular basis of denitrification. Microbiol. Mol. Biol. R..

[70] Tiedje JM, Sexstone AJ, Myrold DD, Robinson JA (1983). Denitrification: ecological niches, competition and survival. Antonie van Leeuwenhoek..

[71] Nelson MB, Berlemont R, Martiny AC, Martiny JBH (2015). Nitrogen Cycling Potential of a Grassland Litter Microbial Community. Appl. Environ. Microb..

[72] Bernhard A (2012). The nitrogen cycle: Processes, players, and human impact. Nature Education Knowledge..

[73] Burris RH, Roberts GP (1993). Biological nitrogen fixation. Annu. Rev. Nutr..

[74] Seitzinger SP (1988). Denitrification in freshwater and coastal marine ecosystems: ecological and geochemical significance. Limnol. Oceanogr..

[75] Van Niftrik L, Jetten MSM (2012). Anaerobic Ammonium-Oxidizing Bacteria: Unique Microorganisms with Exceptional Properties. Microbiol. Mol. Biol. R..

[76] Kuypers M (2003). Anaerobic ammonium oxidation by anammox bacteria in the Black Sea. Nature..

[77] Dalsgaard T, Canfield DE, Petersen J, Thamdrup B, Acuna-Gonzalez J (2003). N-2 production by the anammox reaction in the anoxic water column of Golfo Dulce, Costa Rica. Nature..

[78] Thamdrup B, Dalsgaard T (2002). Production of N-2 through anaerobic ammonium oxidation coupled to nitrate reduction in marine sediments. Appl. Environ. Microb..

[79] Kartal B (2011). Molecular mechanism of anaerobic ammonium oxidation. Nature..

[80] Rosswall T (1982). Microbiological regulation of the biogeochemical nitrogen cycle. Plant Soil..

[81] Lamba S (2017). Organization of biogeochemical nitrogen pathways with switch-like adjustment in fluctuating soil redox conditions. Royal Society Open Science..

[82] Smith VH, Tilman GD, Nekola JC (1999). Eutrophication: impacts of excess nutrient inputs on freshwater, marine, and terrestrial ecosystems. Environ. Pollut..

[83] Guildford SJ, Hecky RE (2000). Total nitrogen, total phosphorus, and nutrient limitation in lakes and oceans: Is there a common relationship?. Limnol. Oceanogr..

[84] Meeks JC, Wycoff KL, Chapman JS, Enderlin CS (1983). Regulation of expression of nitrate and dinitrogen assimilation by Anabaena species. Appl. Environ. Microb..

[85] Yang X (2015). Potential Contribution of Anammox to Nitrogen Loss from Paddy Soils in Southern China. Appl. Environ. Microb..

[86] Ward, B. B., Arp, D. J. and Klotz, M. G. Nitrification. American Society for Microbiology Press (2011).

[87] Frigaard N, Dahl C (2009). Sulfur Metabolism in Phototrophic Sulfur Bacteria[M]. Advances in Microbial Physiology, Poole R K.

[88] Johnson SS, Chevrette MG, Ehlmann BL, Benison KC (2015). Insights from the Metagenome of an Acid Salt Lake: The Role of Biology in an Extreme Depositional Environment. PLoS One..

[89] Friedrich CG, Rother D, Bardischewsky F, Quentmeier A, Fischer J (2001). Oxidation of reduced inorganic sulfur compounds by bacteria: Emergence of a common mechanism?. Appl. Environ. Microb..

[90] Houghton JL (2016). Thiosulfate oxidation byThiomicrospira thermophila: metabolic flexibility in response to ambient geochemistry. Environ. Microbiol..

[91] Jørgensen BB, Nelson DC (2004). Sulfide oxidation in marine sediments: geochemistry meets microbiology. Geological Society of America Special Papers..

[92] Jorgensen BB (1990). A thiosulfate shunt in the sulfur cycle of marine sediments. Science..

[93] Nedwell DB, Abram JW (1979). Relative influence of temperature and electron donor and electron acceptor concentrations on bacterial sulfate reduction in saltmarsh sediment. Microb. Ecol..

[94] Veldhuis MJ, van Gemerden H (1986). Competition between purple and brown phototrophic bacteria in stratified lakes: sulfide, acetate, and light as limiting factors. FEMS Microbiol. Ecol..

[95] Su J (2017). Metagenomic assembly unravel microbial response to redox fluctuation in acid sulfate soil. Soil Biology and Biochemistry..

