# Supplementary material for: Microbial functional genes elucidate environmental drivers of biofilm metabolism in glacier-fed streams
Source: Sci Rep. 2017 Oct 4;7:12668. doi: 10.1038/s41598-017-13086-9 (PMC5627277; doi:10.1038/s41598-017-13086-9)
Supplement: Supplementary file 1 — Supplementary Information [file 41598_2017_13086_MOESM1_ESM.pdf]

Microbial functional genes elucidate environmental drivers of biofilm metabolism in glacier-fed streams

Ze Ren[a], Hongkai Gao[b,c,d], James J. Elser[a], Qiudong Zhao[b]*

[a] *Flathead Lake Biological Station, University of Montana, Polson, MT, 59860, USA*

[b] *State Key Laboratory of Cryosheric Sciences, Northwest Institute of Eco-Environment and Resources, Chinese Academy of Sciences, Lanzhou, 730000, China*

[c] *School of Life Sciences, Arizona State University, Tempe, AZ, 85281, USA*

[d] *School of Geography and Planing, Sun Yat-Sen University, Guangzhou, 510275, China*

[*] *Corresponding author: Email:* hongkai.gao@asu.edu

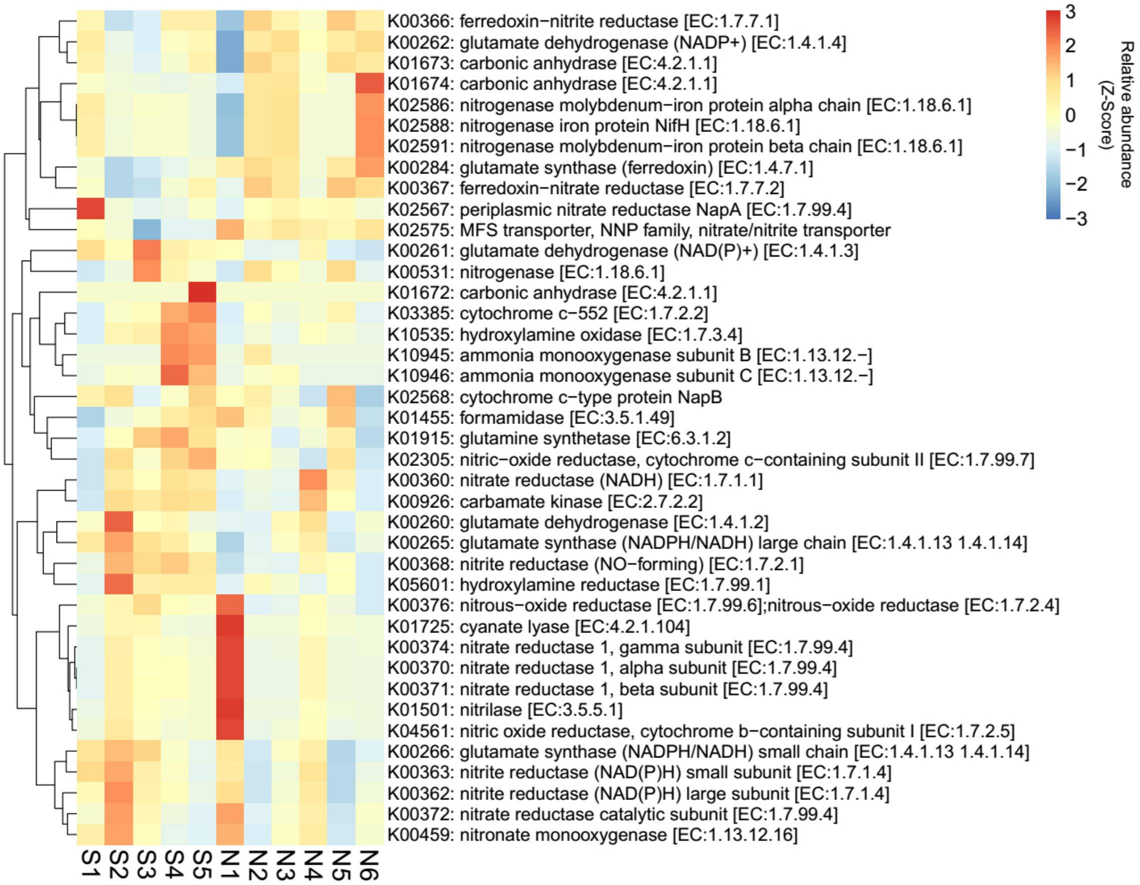

Figure S1 Heatmap showing the relative abundances of PICRUSt predicted genes (y-axis) encoding enzymes that catalyze nitrogen metabolism pathways (ko00910) based on the KEGG database in each sampling site (x-axis).

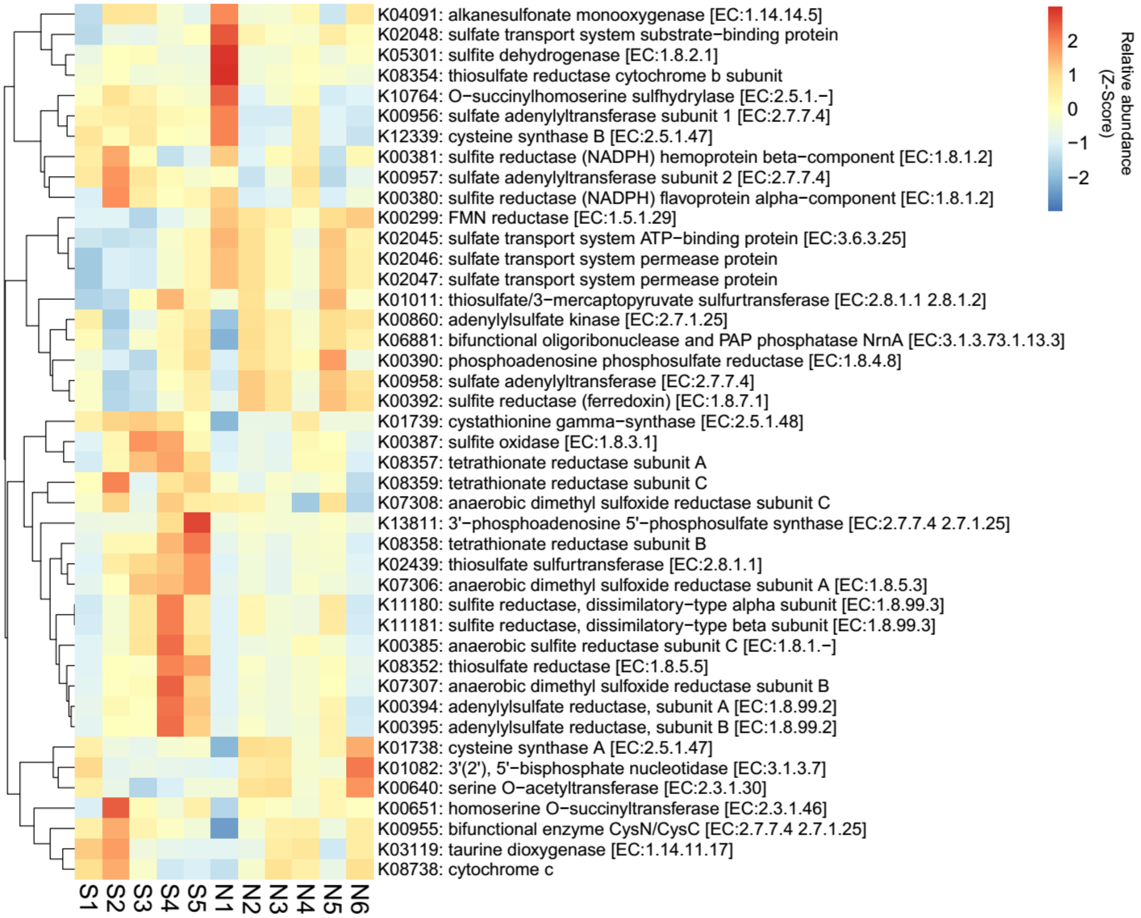

Figure S2 Heatmap showing the relative abundances of PICRUSt predicted genes (y-axis) encoding enzymes that catalyze sulfur metabolism pathways (ko00920) based on the KEGG database in each sampling site (x-axis).

Table S1 Correlate relationships between nitrogen and sulfur metabolism pathways and abiotic environmental factors. P-value was adjusted by Bonferroni correction (abbreviations as in text)

	G_A	G_S	G_D	Altitude	DO	pH	Cond	Temp	TN	NO3	NH4	TP	SRP	DOC
**Nitrogen Metabolism**														
Anammox	**-0.682***	**-0.687***	0.487	-0.250	0.166	**0.732***	0.355	**0.771****	**-0.820****	**-0.851****	-0.399	-0.475	-0.398	**0.866****
Assimilatory Nitrate Reduction	0.099	0.185	0.350	-0.403	0.382	-0.071	0.172	-0.106	**0.607***	**0.638***	0.440	0.263	0.026	-0.581
Denitrification	0.533	0.404	**-0.823****	0.540	-0.493	-0.554	-0.474	-0.350	-0.169	-0.181	-0.377	-0.130	0.130	-0.014
Dissimilatory Nitrate Reduction	0.384	0.280	**-0.724***	0.552	-0.455	-0.436	-0.386	-0.228	-0.159	-0.190	-0.298	-0.122	0.077	0.121
Nitrification	0.578	0.450	**-0.829****	0.504	-0.460	**-0.605***	-0.472	-0.385	-0.118	-0.126	-0.328	-0.089	0.129	-0.083
Nitrogen Fixation	-0.286	-0.164	0.590	-0.481	0.473	0.237	0.354	0.163	0.358	0.380	**0.678***	0.494	-0.080	-0.141
**Sulfur Metabolism**														
Assimilatory Sulfate Reduction	-0.498	-0.383	**0.788****	-0.570	0.559	0.547	0.557	0.421	0.257	0.271	0.325	0.041	-0.089	0.011
Dissimilatory Sulfate Reduction	-0.175	-0.051	0.299	-0.023	0.272	0.183	0.208	0.181	0.284	0.281	0.464	0.27	-0.038	0.133
SOX System	-0.487	-0.565	0.165	-0.066	-0.118	0.444	0.203	0.464	-0.472	-0.538	-0.549	-0.549	-0.165	0.479

Note: "*" indicates level of statistical significance at $P<0.05$, "**" indicates $P<0.01$
